# Empowering Patients and Families Across the Continuum of Care: From Early Risk Detection to Integrated Home Healthcare and Hospital-at-Home Models

**DOI:** 10.1097/jnr.0000000000000773

**Published:** 2026-09-25

**Authors:** Li-Yin CHIEN

Nursing professionals are playing critical roles in the global transition of healthcare paradigms from hospital-centric intervention toward proactive, continuous, and community-embedded care (Markovich et al., 2026). In this issue of *The Journal of Nursing Research*, we bring together rigorous empirical contributions that illuminate how nurses and nurse practitioners (NPs) are empowering patients and families, promoting early risk detection, and seamlessly bridging clinical excellence with home- and community-based care delivery.

Empowering individuals and families to take active roles in self-care and early risk identification is fundamental to sustainable healthcare (Global Self-Care Federation, 2025). Primary prevention and health literacy serve as the first line of defense in community health. Demonstrating this imperative, Lai et al. developed and psychometrically validated the *Human Papillomavirus Prevention Health Literacy Scale for College Students in Taiwan*. Their work provides a reliable, tailored tool that enables healthcare providers to assess health literacy, target educational gaps, and empower young adults to make informed preventive health decisions regarding HPV vaccination and screening.

Beyond primary prevention, empowering self-care requires acknowledging the relational context of health management, especially when dealing with chronic and life-altering conditions. Managing post-stroke recovery, for example, is inherently a journey shared by patients and their family caregivers. Xu et al., 2026 examined the intricate interplay between fear of progression and dyadic self-care among stroke survivors and their spouses. Their findings highlight that psychological burdens heavily influence mutual self-care behaviors, underscoring the necessity for nurse-led family interventions that enhance emotional resilience and collaborative coping in community settings.

As care models decentralize, advanced practice nurses, particularly NPs, play a crucial operational and clinical role in driving patient enablement, satisfaction, and safety across acute and transitional settings. In a multi-hospital study, Chen et al., 2026 evaluated perceived satisfaction, quality of care, and patient enablement among individuals receiving acute care managed by NPs in Taiwan. Their empirical evidence confirms that NP-led care significantly enhances patient enablement, boosting patients' confidence and capacity to independently manage their post-discharge health outcomes.

This capacity-building aspect of nursing is central to community-oriented care. When patients and their family caregivers are empowered with clinical guidance, structured health education, and responsive NP care, they are better able to transition smoothly from acute hospital environments back to their homes and communities, reducing avoidable readmissions and long-term disability.

Looking ahead, the ultimate evolution of community-oriented nursing care resides in the scalable implementation of Home Care and modern Hospital-at-Home models (Leong et al., 2021). The insights gained from assessing health literacy, addressing dyadic psychological distress, and enhancing patient enablement directly inform how acute-level care may be migrated safely into patient homes.

Hospital-at-Home initiatives demand high levels of patient and caregiver engagement, continuous remote monitoring, and advanced clinical management led by specialized nursing teams. By equipping families with self-care capabilities and leveraging the full scope of NP practice, home-based acute and restorative care models can deliver safe, patient-centered, and cost-effective outcomes. As researchers and nurses, our collective mission is to continue generating robust evidence that refines these community and home care paradigms, ensuring high-quality healthcare is accessible wherever the patient resides.
